# Development of Oxytolerant *Salmonella typhimurium* Using Radiation Mutation Technology (RMT) for Cancer Therapy

**DOI:** 10.1038/s41598-020-60396-6

**Published:** 2020-02-28

**Authors:** Shuang Gao, Jong-Hyun Jung, Shun-Mei Lin, A-Yeung Jang, Yong Zhi, Ki Bum Ahn, Hyun-Jung Ji, Jae Hyang Lim, Huichen Guo, Hyon E. Choy, Sangyong Lim, Ho Seong Seo

**Affiliations:** 1 Research Division for Radiation Science, Korea Atomic Energy Research Institute, Jeongeup, Republic of Korea; 2 Department of Molecular Medicine (BrainKorea21 Plus), Chonnam National University Graduate School, Gwangju, Republic of Korea; 3 Department of Radiation Science, University of Science and Technology, Daejeon, Republic of Korea; 4 Department of Internal Medicine, Korea University College of Medicine, Seoul, Republic of Korea; 5 Department of Microbiology, College of Medicine, Ewha Womans University, Seoul, 07985 Republic of Korea; 6 State Key Laboratory of Veterinary Etiological Biology, National Foot and Mouth Disease Reference Laboratory, Lanzhou Veterinary Research Institute, Chinese Academy of Agricultural Sciences, Lanzhou, China

**Keywords:** Targeted therapies, Live attenuated vaccines

## Abstract

A critical limitation of *Salmonella typhimurium* (*S. typhimurium*) as an anti-cancer agent is the loss of their invasive or replicative activities, which results in no or less delivery of anti-cancer agents inside cancer cells in cancer therapy. Here we developed an oxytolerant attenuated *Salmonella* strain (KST0650) from the parental KST0649 (Δ*ptsI*Δ*crr*) strain using radiation mutation technology (RMT). The oxytolerant KST0650 strain possessed 20-times higher replication activity in CT26 cancer cells and was less virulent than KST0649. Furthermore, KST0650 migrated effectively into tumor tissues in mice. KST0650 was further equipped with a plasmid harboring a spliced form of the intracellular pro-apoptotic protein sATF6, and the expression of sATF6 was controlled by the radiation-inducible *recN* promoter. The new strain was named as KST0652, in which sATF6 protein expression was induced in response to radiation in a dose-dependent manner. This strain was effectively delivered inside cancer cells and tumor tissues via the *Salmonella* type III secretion system (T3SS). In addition, combination treatment with KST0652 and radiation showed a synergistic anti-tumor effect in murine tumor model with complete inhibition of tumor growth and protection against death. In conclusion, we showed that RMT can be used to effectively develop an anti-tumor *Salmonella* strain for delivering anti-cancer agents inside tumors.

## Introduction

Spontaneous mutations have been extensively used as sources of novel genetic diversity for selecting new improved organisms^1,2^. After the discovery of X-rays and γ-rays, ionizing radiation (IR)-induced mutation breeding is being widely used to generate genetic variability in various organisms^3,4^. After penetrating inside tissues, γ-rays directly disrupt DNA via deposition of energy, or indirectly via ionization, which generates free radicals from radiolysis of water^5–7^. Even though chemical mutagens and ultraviolet irradiation has been widely used for producing bacterial mutants, deletions and insertions are limitedly introduced in the genome. However, the effects of IR cause various types of random DNA mutations including deletions, insertions, and point mutations during DNA repair. The Food and Agriculture Organization of the United Nations (FAO) and the International Atomic Energy Agency (IAEA) Joint Mutant Varieties Database indicated that over 87% of 3,200 mutant variants in 214 plant species, which were developed using RMT, have been released worldwide^8,9^. However, the use of this technique in bacteria is limited, especially for bacteria that are used for medical applications.

*Salmonella* as a potential anti-cancer tool is valuable for treating cancer because of its selective colonizing and proliferative abilities in nonhypoxic and hypoxic regions^10–12^. However, the high virulence of *Salmonella* limits its application in cancer therapy. Hence, methods of developing attenuated *Salmonella* strains without altering their tumor-targeting ability are being actively investigated. VNP20009, derived from ATCC14028, was genetically attenuated by the deletion of both *msb* and *purI*, which exhibited specific accumulation in various tumor tissues^13,14^. The A1 strain derived from ATCC14028 was constructed by inducing leucine and arginine auxotrophic mutations with a chemical mutagen^15^. This strain selectively targeted the tumor *in vivo* with tumor: liver ratios of 2,000:1, and *Salmonella* colonization was not detected in normal tissue 2 weeks post-injection^16,17^. Avirulent SL∆ppGpp (∆*relA*∆*spoT*) exerted tumor inhibitory effect by recruiting immune cells and inflammatory cytokines^18^. Although certain strains have been attenuated to non-pathogenic levels, their therapeutic activity was modest and transient in the absence of additional manipulation^19^.

As *Salmonella* functions as an outstanding delivery vehicle for transporting various signaling molecules or toxins into the tumor microenvironment, it is obvious that the use of therapeutic cancer agents with *Salmonella* might improve tumor-suppressive effects^20–22^. Various anti-cancer agents were combined with tumor-targeting *Salmonella* strains to improve their effectiveness, such as TNF-related apoptosis-inducing ligand (TRAIL), pore-forming toxin cytolysin A (CylA), and flagellin subunit (FlaB)^22–26^. Despite the considerable progress in *Salmonella*-mediated cancer therapy, the adverse effects due to poor and inappropriate expression of therapeutic molecules in tumor or normal cells are yet to be overcome. Thus, the application of *Salmonella* harboring controllable radiation inducible promoters (RIPs) may improve the anti-tumor effect of therapeutic *Salmonella* strains by delivering anti-cancer substances in a precise temporal and spatial manner.

In this study, we developed an oxytolerant, hyper-attenuated, and tumor-targeting *Salmonella* strain KST0650 by accelerating its mutation using gamma irradiation. KST0650 has the higher ability to invade and replicate in tumor cells with significantly diminished toxicity. KST0650 was further modified to deliver the anti-cancer molecule, spliced ATF6, under the control of the radiation inducible promoter *recN* (KST0652). Finally, in combination with radiotherapy, this newly developed attenuated mutant strain KST0652 showed complete suppression of tumor growth and protection against death in mice.

## Results

### Construction of an oxytolerant *Salmonella* strain (KST0650) using selectively irradiation

**Figure 1:**
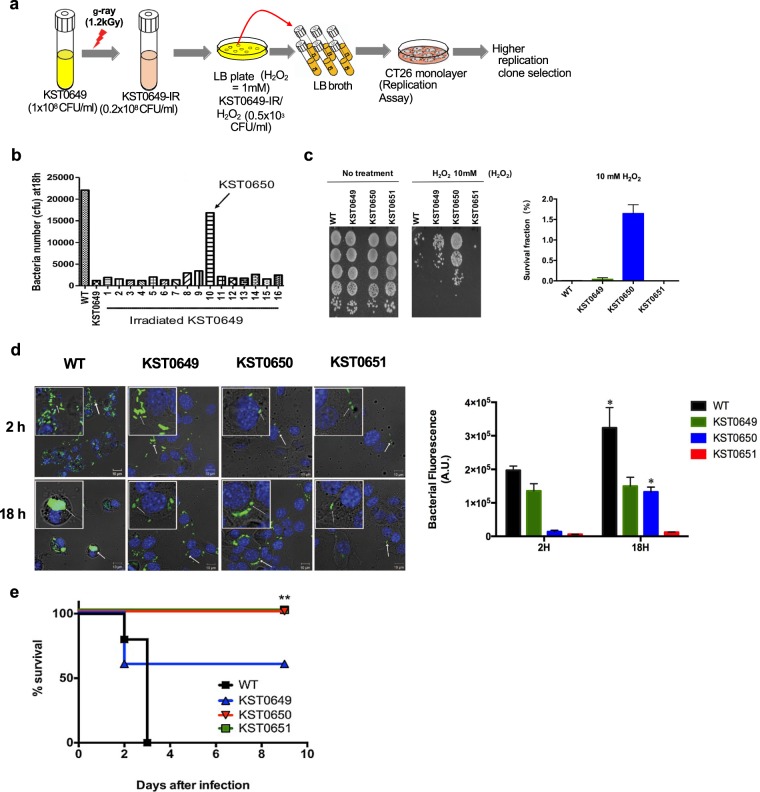
Construction of oxytolerant cancer-targeting *Salmonella* strain using radiation mutation breeding technology. **(a)** Schematic showing the procedure for isolating the *Salmonella* mutant, which was highly oxytolerant and cancer-targeting. The attenuated *Salmonella* strain, KST0649, was irradiated with a sub-lethal dose of γ-radiation (1.2 kGy) (KST0649-IR), followed by selection of the mutant strain, which was resistant to H_2_O_2_ (1 mM) (KST0649-IR/H_2_O_2_) and showed higher replicative ability in CT26 cancer cells. **(b)** KST0650 has the highest replication ability in CT26 cells than its parent strain (KST0649) and other mutants. Gentamicin protection assay was used to determine the replication ability (18 h) of irradiated KST0649 mutants in CT26 cells. **(c)** KST0650 is an oxytolerant strain. WT, KST0649, KST0650, and KST0651 strains were incubated with 10 mM H_2_O_2_ for 1 h, followed by spotting on LB agar plate. Survival fraction were measured by comparing with 0 mM H_2_O_2_. **(d)** Immunofluoresence assay, followed by confocal microscopy, was used to visualize the invasion (2 h) and replication (18 h) of the *Salmonella* strains. The nuclei of CT26 cells were stained with DAPI (blue) and *Salmonella* strains were stained with FITC-conjugated anti-*Salmonella* rabbit IgG (green). White arrows indicate *Salmonella* strain. Representative images are shown; original magnification, 80×; magnification in the white box, 320×. Changes in bacterial fluorescence signals in CT26 cells. Data are fluorescence intensities (a.u.) ± SEM of *Salmonella* from three independent photos and asterisks (*) indicate significant difference between 2 H and 18 H group (*P < 0.05). **(e)** KST0650 is a highly attenuated *Salmonella* strain in mice. BALB/c mice (n  =  5) were injected i.p. with 1 × 10^6^ cfu *Salmonella* strain, and mice survival were monitored every 12 h for 9 days.(**P < 0.01).

The replication ability of *Salmonella* in intracellular vacuoles is dependent on several defense mechanisms, including resistance to vacuole stress conditions, such as hydrogen peroxide^27,28^. However, most attenuated *Salmonella* strains, including KST0649 (∆*ptsI*∆*crr*) and KST0651 (∆*relA*∆*spoT*)^29,30^, cannot replicate in macrophages and epithelial cells, which limits their application in cancer therapy (Fig. S1). To select *Salmonella* strains with high replication ability to develop efficient cancer therapeutic *Salmonella* strains, the attenuated *Salmonella* vaccine strain (KST0649) with defective PTS sugar uptake system^30^ was mutated using γ-irradiation, followed by selection of the oxytolerant strains on Luria Bertani (LB) plate containing hydrogen peroxide (H_2_O_2_; 1 mM) (Fig. 1a). Lethal dose 80 (LD_80_) of γ-radiation was selected as it induced highest mutation ratio in *Salmonella* (data not shown). The surviving *Salmonella* strains were randomly selected and their intracellular replication ability in mouse colon cancer cell line (CT26) was determined. Approximately 0.08−0.2 × 10^8^ cfu/ml (KST0649-IR) bacteria were recovered from 1 × 10^8^ cfu/ml KST0649 after γ-irradiation. Only 0.5 × 10^3^ cfu/ml KST0649-IR/H_2_O_2_ (oxytolerant irradiated KST0649) survived on the H_2_O_2_-LB plate after irradiation. It is noteworthy that no colonies were detected on the H_2_O_2_-LB plate inoculated with non-irradiated KST0649 (data not shown). To determine whether the increase in oxytolerance results in higher survival and replication in CT26 cells, randomly selected oxytolerant strains were used to infect CT26 cells and their survival and replication at 18 h post-infection was compared with those of the parent strain (KST0649) and wild type (WT, LT2). Most of the selected mutants showed similar or slightly higher levels of replication rate compared to the parent strain (KST0649) in CT26 cells; however, colony #10 showed approximately 20-times higher replication rate (Fig. 1b). This highly replicating oxytolerant mutant strain was selected for the subsequent studies and named as KST0650. To confirm whether KST0650 has higher resistance to oxidative stress, it was exposed to different concentrations of H_2_O_2_ in LB broth, followed by determination of the survival ratio. The survival ability of KST0650 was at least 100, 10, and 1000 times higher than those of the WT, KST0649, and KST0651, respectively, at 10 mM H_2_O_2_ (Fig. 1c).

To visualize the invasion and replication ability of KST0650 in CT26 cells, intracellular *Salmonella* were stained with FITC-labeled *Salmonella*-specific IgG and visualized using confocal microscopy (Fig. 1d). The fluorescent signals from the CT26 cells infected with the WT strain were significantly stronger than those from the other mutant strains at both 2 h and 18 h. The extent of KST0650 infection was lower than that of KST0649 at 2 h, but the intracellular bacterial population of KST0650 increased more obviously than that of KST0649 at 18 h. This suggested that KST0650 survives and replicates in the CT26 cells more efficiently than the parent strain.

The toxicity of cancer-targeting *Salmonella* is another important criterion for considering *Salmonella*-mediated cancer therapy. To determine whether KST0650 was sufficiently attenuated for use as a cancer therapy strain, its virulence was compared with those of WT, KST0649, and KST0651 (Fig. 1e). Mice (n = 5 per group) were intraperitoneally (i.p.) injected with 1 × 10^6^ cfu of WT or mutant strains and their survival was monitored. All mice infected with the WT strain died within 3 days, and 40% of the KST0649 infected mice succumbed to disease. However, all mice infected with KST0650 or KST0651 exhibited 100% survival for more than 9 days. The LD_50_ value (LD_50_ = 2 × 10^7^ cfu) of KST0650 was similar to that of the well-characterized cancer-targeting *Salmonella* strain KST0651 (LD_50_ = 0.4–2 × 10^7^ cfu), which suggested that KST0650 had similar or slightly lower virulence than KST0651 (Table S1)^20–22,31^. Based on this result, 1 × 10^6^ cfu i.p. was selected as the treatment dose of KST0650 for the subsequent experiments. Taken together, mutant *Salmonella* strain KST0650 developed using RMT from the attenuated strain KST0649 was able to survive within the hostile environments better than KST0649 due to high resistance to oxidative stress, while it showed significant reduction in virulence in a mouse model.

### Comparative genome analysis of WT and KST0650 strains

To analyze the location of mutations in KST0650, the complete genome of KST0650 was sequenced and compared to that of WT as a reference. As shown in Tables 1 and S2, there were 89 genetic alterations, including 10 deletions, 30 insertions, 16 inversions, and 33 substitutions. Overall, the genome size was 51 nucleotides shorter than that of WT (strain LT2) due to deletion and insertion mutations. Forty percent of the deletions (4 of 10) and 87% of the insertions (26 of 30) constituted only one nucleotide addition or loss, which resulted in frameshift mutations. Forty percent of the deletions (4 of 10) and 10% of the insertions (3 of 30) were non-frameshift mutations, in which a multiple of three nucleotides were inserted or deleted. Among the point mutations, eight mutations were detected in non-coding regions (NCR), and 25 substitutions and 16 inversions were present in the coding regions. Among the 25 substitutions, 17 mutations were non-synonymous substitutions, including 14 missense and 3 non-sense mutations. All inversions specifically occurring from CG to GC resulted in 16 missense non-synonymous substitutions. These high non-synonymous mutation rates revealed that RMT was a strong driving force for bacterial evolution. All or the part of these alterations of nucleotides and amino acids might together affect oxytolerance and attenuation of KST0650.

**Table 1:** Summary of the mutational spectrum observed in oxytolerent KST0650 strain.

Types		Deletion	Insertion	Inversion	Substitution		Total
					Transversion	Transition	
Non-coding region		4 (1^a^)	17 (5^b^)	0	5	3	29
Coding region	Silent	—	—	-	2	6	8
	Mis-sense	—	—	15	5	9	29
	Non-sense	—	—	1	1	2	4
	Frameshift	3	10 (6^c^)	—	—	—	13
	Non-frameshift	3	3	—	—	—	6
Total		10	30	16	13	20	89

a = Number of non-frameshift in non-coding region (NCR).

b = mutation in sequence of pseudogene.

c = Number of truncation caused by frameshift.

### Effective cancer targeting by KST0650

**Figure 2:**
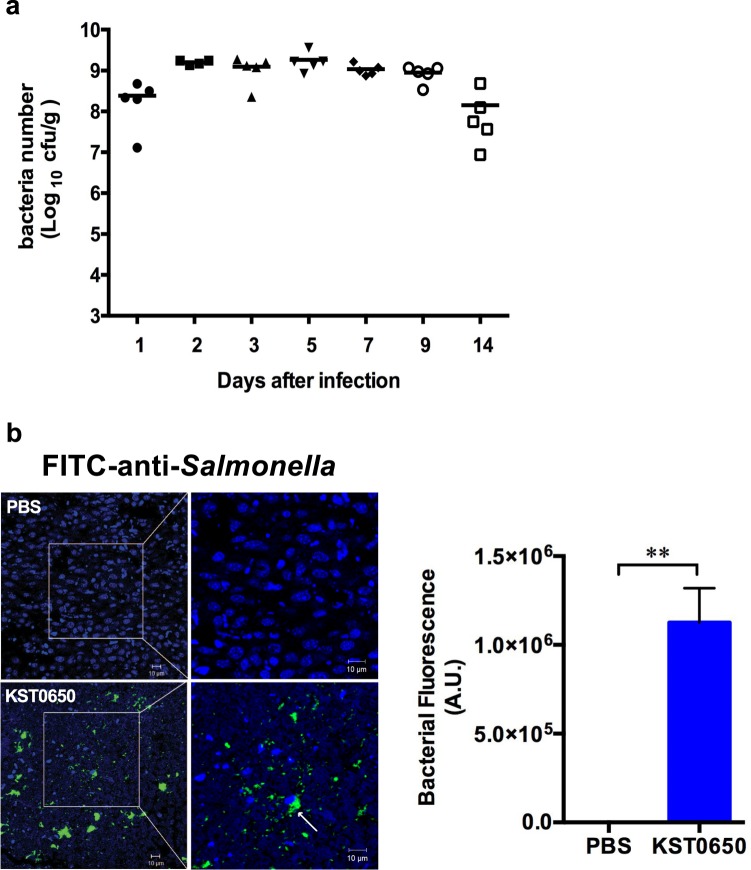
Effective tumor targeting by KST0650. CT26 cancer cell-bearing mice were injected i.p. with 1 × 10^6^ cfu KST0650. **(a)** The numbers of KST0650 in CT26 tumors of BALB/c mice were calculated for 14 days. **(b)** Immunofluoresence assay was used to visualize the tumor-targeting ability of KST0650 in CT26 tumor tissues. The nuclei of CT26 tumor cells were stained with DAPI (blue) and the *Salmonella* strains were stained with FITC-conjugated anti-*Salmonella* rabbit IgG (green). White arrows indicate KST0650 strain. Representative images are shown; original magnification in the left panel, 40×; original magnification in the right panel, 80×. Bacterial fluorescence signals in tumor. Data are fluorescence intensities (a.u.) ± SEM of KST0650 and asterisks (*) indicate significant difference between each group (**P < 0.01).

To investigate the tumor-targeting efficacy of KST0650, CT26 tumor-bearing mice were infected with 1 × 10^6^ cfu KST0650, followed by measurement of bacterial number in tumor tissue and normal organs. As shown in Figs. 2a and S2, the number of KST0650 in tumor tissues increased rapidly, and was 1,000–100,000 fold higher than that in other normal organs, such as liver, spleen, kidney, lungs, and blood at 1-day post-infection. The bacterial load was maximum in tumor tissues at day 2 (approximately 10^9^ cfu/g tissue weight) and maintained at a relatively constant level for more than 14 days. In contrast, all bacteria disappeared or rarely detected at day 3 (blood) or day 14 (spleen, lung, liver, and kidney) in normal organs. To visualize the tumor-targeting bacteria *in vivo*, tumor tissues were stained with FITC-conjugated anti-*Salmonella* antibody and visualized using confocal microscopy at day 28 after KST0650 inoculation. Large amounts of KST0650 fluorescent signals were dispersed in tumor tissues, and some signals were located adjacent to the nuclei (Fig. 2b), indicating that KST0650 efficiently targeted tumors and invaded CT26 tumor cells in mice. The direct inhibitory effect of KST0650 on tumor size and mice survival were also assessed *in vivo* (Fig. S3). Although KST0650 preferentially localized and multiplied in tumor tissues for longer time, there were no obvious differences in tumor size and survival rate between the groups inoculated with phosphate buffered saline (PBS) and KST0650, respectively. These results indicated that KST0650 cannot directly suppress tumor growth.

### Production and secretion of sATF6 using a radiation-inducible vector in KST0650

**Figure 3:**
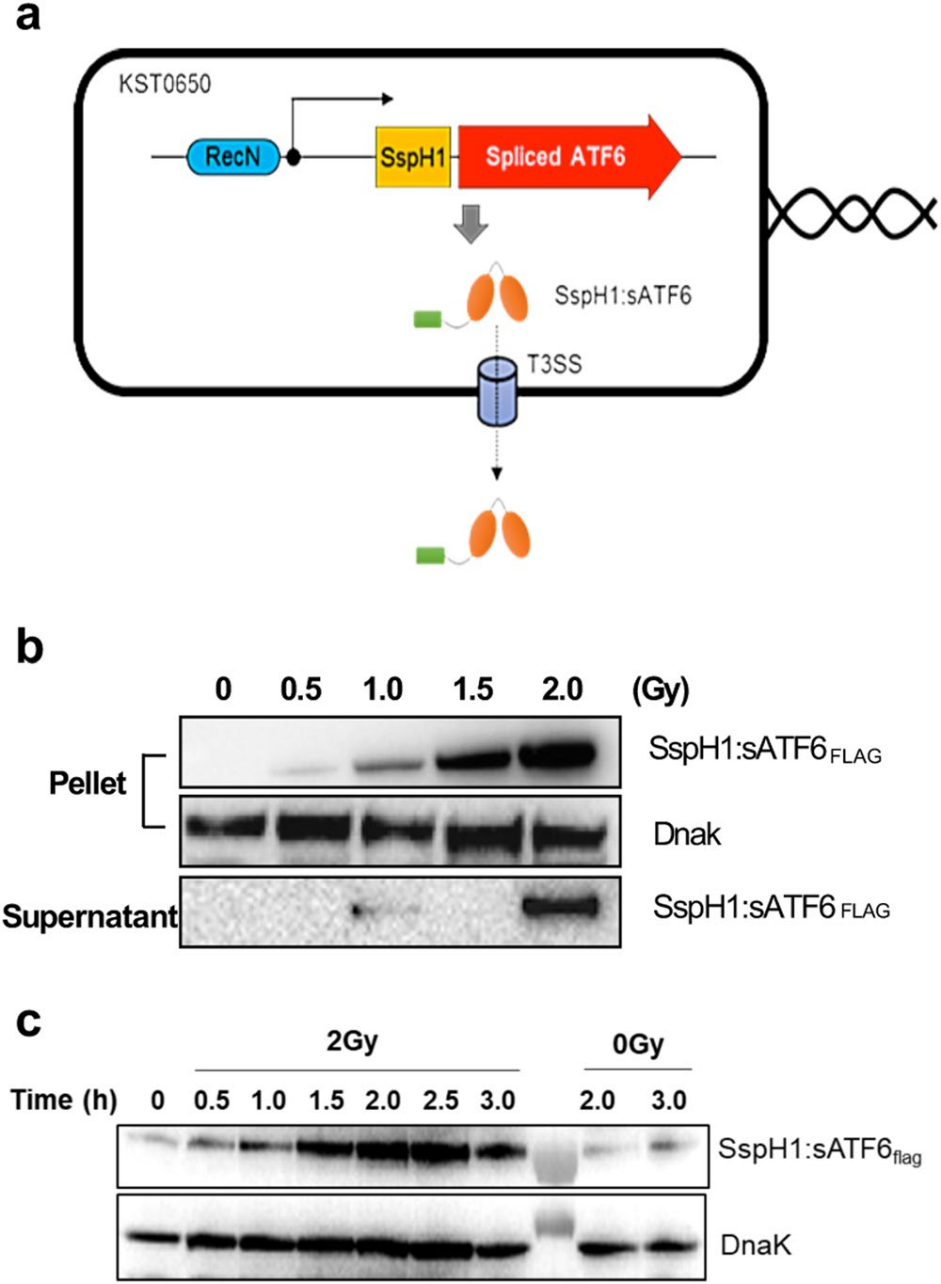
Expression and secretion of SspH1:sATF6 fusion protein in KST0650. **(a)** Schematic map of radiation-inducible SspH1:sATF6 expression and export system (pRS-sATF6). **(b)** KST0652-expressing SspH1:sATF6 was induced with the indicated dose of γ-radiation, followed by incubation for 2 h at 37 °C. SspH1:sATF6 protein level was determined using western blot analysis with anti-FLAG antibody in the bacterial lysate and bacterial culture supernatant. DnaK was detected and used as the loading control. **(c)** Expression of SspH1:sATF6 in KST0652 was measured using western blot analysis at the indicated time points after 2 Gy of γ-radiation. Full-length blots are presented in Supplementary information.

Although KST0650 exhibited low tumor-suppression potency, it replicated efficiently in tumor cells, unlike the well-studied cancer-targeting strain KST0651. To take advantage of the high replication ability of KST0650, an endothelium reticulum stress (ERS)-related gene encoding spliced activating transcription factor 6 (sATF6), which shows low expression in colon cancer tissues compared to the corresponding tumor-adjacent tissues and triggers cell apoptosis by inducing downstream molecules like Bax, Bak, and CHOP, was introduced into KST0650^32–37^. A new plasmid encoding FLAG-tagged mouse sATF6 (sATF6_FLAG_) under the *Salmonella recN* promoter, one of the major radiation-responsive promoters, was constructed to express sATF6 in KST0650 (Fig. 3a)^38,39^. In addition, sATF6 was fused with a well-known signal sequence of SspH1_1–140_, which enabled secretion of the fused protein via the *Salmonella* type III secretion system (T3SS)^40,41^. KST0650 harboring pRS-sATF6_FLAG_ was named strain KST0652.

To confirm the expression and secretion of sATF6_FLAG_ in KST0652 in response to γ-radiation, KST0652 was irradiated in mid-log phase, and the expression and secretion of sATF6_FLAG_ were determined using western blot analysis. SspH1:sATF6_FLAG_ was expressed effectively in KST0652, and the expression level was increased markedly by γ-radiation in a dose-dependent manner (Fig. 3b). The expression level of SspH1:sATF6_FLAG_ was maximum at 2.5 h post-irradiation (Fig. 3c). SspH1:sATF6_FLAG_ secretion was also assessed in the culture supernatant of KST0652. SspH1:sATF6_FLAG_ was barely detected in the culture supernatants of KST0652 under the no or low dose of γ-radiation; however, high SspH1:sATF6_FLAG_ level was detected in the culture supernatant following irradiation with 2 Gy γ-rays (Fig. 3b). These results confirmed that sATF6 protein expression was regulated by the *recN* promoter in response to γ-radiation, and it was secreted efficiently from KST0652.

### Proapoptotic effect of sATF6 on CT26 cells

To determine the cytotoxic activity of SspH1:sATF6_FLAG_ on CT26 cancer cells, proliferation and apoptosis of CT26 cells were measured after transfecting the SspH1:sATF6_FLAG-_expressing plasmid (pcDNA3.1( + )-SspH1:sATF6_FLAG_) transiently into CT26 cells. The proliferation of cells transfected with the SspH1:sATF6_FLAG_ expressing plasmid was lower than that of the control group (Fig. S4a), and the total apoptotic and necrotic rates of sATF6-expressing cells increased to 48.55% and 13.69%, respectively, which were significantly higher than that of the non-transfected (13.44% and 4.94%) or control plasmid (22.93% and 4.67%)-transfected cells (*P* < 0.01) (Fig. S4b). In addition, sATF6 overexpression in CT26 cells significantly enhanced the expression of *Chop* and spliced *Xbp-1* (*sXbp-1*) mRNAs, the downstream effectors of sATF6, which interact with the members of the Bcl-2 family (Fig. S4c)^42,43^.

**Figure 4:**
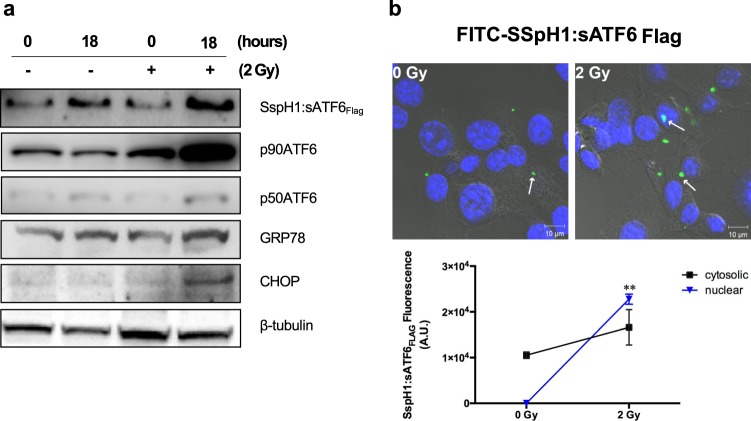
Endoplasmic reticulum stress (ERS)-related protein expression in CT26 cells infected with KST0652. The CT26 cell monolayer was infected with KST0652 (MOI = 100) and irradiated with 2 Gy of γ-radiation at 2 h post-infection. **(a)** The expression levels of SspH1:sATF6_FLAG_, p90ATF6, p50ATF6, GRP78, and CHOP proteins were detected using western blot analysis. β-tubulin was used as the loading control. Full-length blots are presented in Supplementary information. **(b)** SspH1:sATF6_FLAG_ was detected in KST0652-infected CT26 cells after 18 h post-irradiation. Immunofluorescence assay, followed by confocal microscopy, was used to visualize the location and expression of SspH1:sATF6_FLAG_ in CT26 cells. The nuclei of CT26 cells were stained with DAPI (blue), and SspH1:sATF6_FLAG_ were stained with FITC-conjugated anti-FLAG mouse IgG (green). White arrows indicate SspH1:sATF6_FLAG_ protein. Representative images are shown; original magnification, 100×. Changes in fluorescence signals of SspH1:sATF6_FLAG_ in CT26 cells. Data are mean fluorescence intensities (a.u.) ± SEM from three independent photos and asterisks (*) indicate significant difference between each group (** *P* < 0.01).

To further investigate whether the expression and release of SspH1:sATF6_FLAG_ from KST0652 activates unfolded protein response (UPR) in CT26 cells, CT26 monolayers were infected with γ-irradiated KST0652 and the levels of ERS-related proteins were determined (Fig. 4a). Western blot analysis showed that SspH1:sATF6_FLAG_ expression was detected in CT26 cells, the levels of which increased after γ-irradiation as expected. This was also marked by considerable increase in the expression of p90ATF6 and p50ATF6 proteins in the irradiated cells, and GRP78, a master initiator of early ERS, was up-regulated. In addition, the expression of proapoptotic protein CHOP increased markedly in KST0652-infected cells exposed to γ-radiation, suggesting that CHOP expression was likely to be activated by the combined effect of radiation and KST0652. As CHOP transcription is regulated by sATF6, the expression and localization of SspH1:sATF6_FLAG_ in CT26 cells were determined using FITC-conjugated anti-FLAG antibody and confocal microscopy at 18 h after irradiation. The SspH1:sATF6_FLAG_ signal was weak in CT26 cells without γ irradiation, but stronger signals were detected in the nucleus after exposure to 2 Gy of γ-radiation, suggesting that SspH1:sATF6_FLAG_ translocated to the nucleus to become functional (Fig. 4b).

### KST0652 and γ-radiation synergistically suppress tumor growth

**Figure 5:**
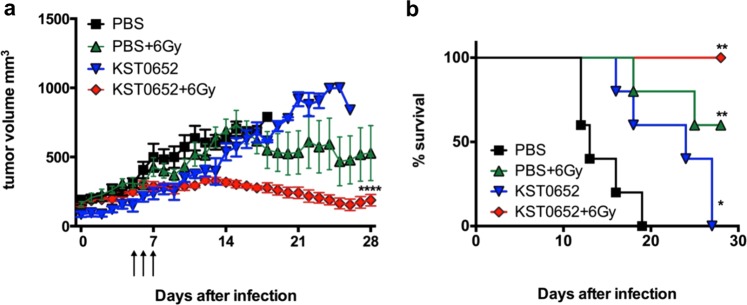
Suppression of CT26 tumors with a combination of KST0652 and γ-radiation. CT26 tumor-bearing mice were injected with PBS or KST0652, followed by radiotherapy with 2 Gy of γ-radiation at days 5, 6 and 7. **(a)** Tumor volumes were measured daily for 28 days. Data represent mean ± SEM and asterisks (*) indicate significant difference between each group (****P < 0.0001). **(b)** Mice survival analysis was based on follow-up until tumor volume exceeded 1,000 mm^3^ (*P < 0.05; **P < 0.01).

To investigate the tumor suppressive efficacy of KST0652, CT26 tumor-bearing mice were i.p. inoculated with PBS or 1 × 10^6^ cfu of KST0652. Compared to the PBS injection, KST0652 injection cannot reduce the tumor size, but the survival of KST0652-injected mice was significantly higher than that of the PBS-injected mice (Fig. 5a,b). After five days, at which time KST0650 was cleared from blood and reached maximal level in the tumor tissues, the subgroups received fractional dose of γ-radiation (2 Gy/day) for 3 days, and changes in tumor size were measured. The control group (PBS-injected mice) showed mild tumor growth inhibiting effect after exposure to γ-radiation. However, the irradiated KST0652-injected mice showed complete suppression of tumor growth (Fig. 5a). In addition, γ-radiation exposure alone increased survival from 0% to 60% in mice injected with PBS, but completely protected KST0652-injected mice from death (Fig. 5b). Thus, KST0652 and radiation exerted a synergistic effect on tumor suppression.

Taken together, these results indicated that a newly developed mutant *Salmonella* strain, KST0652, efficiently targeted tumor tissues and replicated profusely *in vivo*. It completely inhibited tumor growth and protected mice from death when combined with γ-radiation therapy.

## Discussion

In this study, we developed a new tumor targeting *Salmonella* strain (KST0650) by introducing random mutations using RMT. The selected strain KST0650 showed lower toxicity and higher replication ability than its parent strain (KST0649), probably due to its oxytolerance^44^.

To the best of our knowledge, γ-radiation has not been widely used in bacterial mutation breeding because, unlike plant breeding, it requires a high-dose radiation facility, and all mutations have to be detected and selected painstakingly only at the phenotypic level. However, recent advances in large-scale genomic analysis techniques have enabled easy analysis of the effects of radiation and the location of mutations in the bacterial genome. In this study, we analyzed the genome of KST0650 and compared it with that of its parent WT strain (LT2), which resulted in the identification of 89 mutations. The mutations occurred uniformly throughout the genome of KST0650, but the insertions, deletions, and single nucleotide point mutations were mostly concentrated in G and C, rather than in T and A, indicating that radiation-induced mutations may target specific sequences of the bacterial genome. We have not investigated how these mutations cause attenuation and oxytolerance in KST0650. The overall complex interplay between multiple radiation-induced mutations might cause shift in phenotypes, which might be more stable in a revertant than in a mutant generated using genetic methods. In addition, as γ-radiation rapidly and efficiently improve specific properties of bacteria, it can be applied in microbe-based industries.

Although KST0650 was highly attenuated, it preferentially accumulated in tumor cells *in vivo*, which was almost comparable with the levels of other well-known cancer targeting strains, such as VNP20009, SL∆ppGpp (∆*relA*∆*spoT*), and A1-R^13,18,45^. Despite it could effectively target, colonize, invade tumors, and reduce tumor growth rate slightly as compared with PBS only, which helps prolong mice survival, its tumor-curing effect was not observed obviously in a tumor-bearing mouse model, suggesting that KST0650 monotherapy does not show anti-tumor effect effectively. To improve the tumor inhibitory effect of KST0650, it was equipped with the sATF6 protein, which can suppress tumor growth by inducing apoptosis. This newly developed strain KST0652 that expressed and secreted sATF6 in response to γ-radiation decreased tumor growth significantly when combined with radiation therapy. Furthermore, combination therapy of KST0652 and radiation synergistically increased mice survival. As low-dose radiation can enhance antigen presentation on MHC class I of irradiated tumor cells, the irradiated tumor cells containing intracellular *Salmonella* present not only more tumor antigens, but also more *Salmonella* antigens, which leads to effective accumulation of cytotoxic T lymphocytes in both irradiated and *Salmonella*-infected cells^46,47^. In addition, both *Salmonella* and radiation can trigger the secretion of pro-inflammatory cytokines, which can recruit immune cells in tumor tissues^18^. Further studies are required to clarify the molecular mechanisms underlying the synergistic anti-tumor effect of low-dose radiation and *Salmonella*-infection.

Another limitation of *Salmonella* cancer therapy is the systemic toxicity of cytotoxic proteins secreted by *Salmonella* to normal cells. One way to circumvent it is using a site-specific inducible gene expression system. Hypoxia-inducible promoter (HIP-1)-driven reporter gene expression was up to 15-fold higher than that from a constitutively expressing promoter when tumors were hypoxic^48^. However, the promoter cannot be activated in small (nonhypoxic) tumors as well as during metastasis. Another strategy involves the use of radiation-inducible promoters, which was utilized in this study. As radiation can penetrate tissue and cause DNA damage, it might trigger the expression of several DNA repair genes of *Salmonella* in tumor tissues, including *recA*, *recN*, *sulA*, and *dinF*^38,39,49^. In addition, combination therapy involving tumor-seeking *Salmonella* and radiotherapy may induce synergistic tumor suppression and less cytotoxicity to normal cells^46,50,51^. Previously, the *recA* promoter was used for the combination therapy of *Salmonella* inducible systems and radiation; however, the basal activity of the *recA* promoter is high even under normal conditions, which may be toxic to normal cells. In contrast, although RecN is expressed after RecA activation, the basal level of *recN* transcription is considerably lower, and its response to γ-radiation seems to be slightly higher than that of the *recA* promoter^52,53^. Hence, we used the *recN* promoter to control the expression of sATF6 in KST0652, and observed that the expression of SspH1:sATF6 was effectively controlled by the *recN* promoter in response to radiation. Radiation dramatically increased the transcription and translation of SspH1:sATF6 in a dose-dependent manner.

In conclusion, to the best of our knowledge, this is the first report showing that bacteria can be easily and successfully engineered using RMT to generate, for example, cancer-targeting *Salmonella*. This technique can be applied for the development of new advanced bacterial strains for producing vaccines, drugs, and bioenergy, and also for bioremediation. In addition, since this new cancer-targeting *Salmonella* (KST0652) can deliver cytotoxic proteins in tumor cells with a controllable promoter, the system can be used not only for cancer therapy, but also for clinical diagnosis of cancer and as a cancer vaccine.

## Methods

### Bacterial strains and growth condition

The bacterial strains used in this study are listed in Table S3. *Salmonella* or *Escherichia coli* (*E. coli*) strains were grown in LB broth (Becton Dickinson; Franklin Lakes, NJ, USA) or agar plates at 37 °C. Antibiotics were supplied, if required (chloramphenicol, 34 μg/ml; kanamycin, 50 μg/ml; ampicillin, 100 μg/ml). KST0649 and KST0651 strains were constructed using the λ red recombination system as described previously^54^. The KST0650 strain was isolated from its parent strain (KST0649) using RMT. In Brief, KST0649 was cultured in LB broth (OD_600_ = 0.5), followed by irradiation using a Co-60 gamma irradiator (Advanced Research Technology Institute; JeonEupSi, Korea) for 0.5 h (1.2 kGy). Irradiated KST0649 was spread on LB agar with 1 mM H_2_O_2_, followed by incubation for 48 h at 37 °C. The colonies were randomly selected and cultured in LB broth for 24 h and the high H_2_O_2_-resistant strains were selected.

### Hydrogen peroxide sensitivity assay

*Salmonella* strains were grown to OD_600_ = 0.3 at 37 °C, followed by treatment with 10 mM H_2_O_2_ for 1 h. Aliquots were taken directly before or after H_2_O_2_ treatment, serially diluted in PBS, and plated onto LB agar plates to count bacterial number.

### Analysis of bacterial invasion and replication in CT26 cells

Mouse colon carcinoma cell line CT26 was obtained from the Korean Cell Line Bank (KCLB; Seoul, Korea) and grown in the high-glucose Dulbecco’s modified Eagle’s medium (DMEM) (Gibco; Waltham, MA, USA) containing 1% penicillin-streptomycin mixture (Lonza; Walkersville, MD, USA) and 10% fetal bovine serum (Gibco) at 37 °C in the presence of 5% CO_2_. The invasive and replicative ability of *Salmonella* strains in CT26 cells were measured using a previous method^55^. Briefly, CT26 cells (1 × 10^5^ cells per well) were seeded onto 24-well plates and incubated for 24 h. *Salmonella* strains were harvested at the stationary phase and added onto the cell monolayer at multiplicity of infection (MOI) of approximately 10. After incubating for 2 h at 37 °C, the cells were washed thrice with PBS (Lonza). Media was changed with 100 μg/ml gentamicin-supplemented DMEM to remove extracellular bacteria. The cells were incubated for additional 2 h for invasion or 18 h for intracellular replication at 37 °C. Subsequently, the cells were washed thrice with PBS, lysed with 0.5% Triton X-100, and serial dilutions of the lysate were plated on LB agar to assess CFU/ml.

### High-throughput sequencing using an illumina platform

To investigate nucleotide substitutions, deletions, and insertions in the attenuated strain KST0650, its genome was sequenced using Illumina HiSeq 2000 (150 bp paired-end) with 825.98–fold coverage. The total length of read bases was 4,088,887,030 bp, which covered 99.98% length of the WT LT2 strain. The raw reads from the LT2 genome were mapped and aligned to the reference genome sequence using Burrows-Wheeler aligner (BWA-0.7.12) and Picard. Next, the genetic variants were detected using SAMTools (ver. 1.2). All coding variants were identified based on the ORFs of *Salmonella enterica* subsp. *enterica* serovar typhimurium str LT2 (NC_003197) in the National Center for Biotechnology Information (NCBI) database.

### Analysis of bacterial internalization and sATF6 expression using confocal microscopy

The CT26 cells were infected with *Salmonella* strains for 2 or 18 h, the cells were fixed with 4% paraformaldehyde for 15 min, washed with PBS for 5 min, and permeabilized with 0.5% Triton X-100. For the *in vivo* assay, formalin-fixed tumor sections from CT26 tumor-bearing *Salmonella*-infected mice were embedded in paraffin and permeabilized with 0.5% Triton X-100. Intracellular *Salmonella* and nuclei of tumor slices were stained with FITC-conjugated anti-rabbit *Salmonella* IgG (Abcam; Cambridge, UK) and DAPI (Life technologies; Carlsbad, CA, USA), respectively, at 4 °C for 12 h. Expression of SspH1:sATF6_FLAG_ was detected using the FITC-conjugated anti-FLAG mouse IgG (Sigma-Aldrich). The slides were washed with PBS and mounted with mounting medium (Dako; Carpinteria, CA, USA). Images were captured using a laser scanning confocal microscope LSM510 (Carl-Zeiss; Jena, Germany). Average fluorescent signal of three independent photos were calculated using ImageJ software (www.imagej.nih.gov).

### Animal experiments

All animal experiments were approved by the committee on the Use and Care of Animals at Korea Atomic Energy Research Institute (KAERI) and were performed according to the accepted veterinary standards. Six-week-old male BALB/c mice were purchased from Orient Bio. (Suwon, Korea). Mice (n = 5 per group) were intraperitoneally (i.p.) inoculated with WT strain LT2, KST0649, KST0650, or KST0651 strains, and their survival was monitored for 9 days. The tumor-bearing mouse model was generated using a previous method^56^. CT26 cells were subcutaneously (1×10^6^ cells suspend in 50 μl PBS) injected on the right dorsum of BALB/c mice. Mice were randomly divided into each group depended on the body weight, and there is no blinding. Mice were used for the following experiments when the tumor volumes were approximately 100−120 mm^3^. Tumor volume was calculated using the following formula:

$${\rm{Tumor}}\,{\rm{volume}}\,({{\rm{mm}}}^{3})=\frac{(length)\times (width)\times (height)\times \pi }{6}$$

The biodistribution of KST0650 in tumor tissue was calculated as described previously^57^. Briefly, KST0650 were i.p. injected at a dose of 1 × 10^6^ cfu into tumor-bearing mice, which were sacrificed at scheduled time points (1, 2, 3, 5, 7, 9 and 14 days) post-injection. Tumor tissue and normal organs (kidney, lung, spleen, liver, and blood) were collected and homogenized on ice using a homogenizer (IKA; Albany, NY, USA). Serial dilutions of tissue homogenates were dotted on the LB agar plates and incubated overnight to enumerate bacterial number (cfu/g). To investigate the synergistic anti-tumor effects of radiation and *Salmonella* on tumor progress, tumor-bearing mice were injected with *Salmonella* strains or PBS. Subgroups of mice were exposed to single dose of 2 Gy γ-radiation 5, 6, and 7 days post-injection. Tumor volumes and mice survival were observed daily. Mice were sacrificed when tumor volume exceeded 1,000 mm^3^. Follow-up was limited to 4 weeks.

### Construction and transfection of plasmids

The plasmid and primers used in this study are listed in Tables S3 and S4. For constructing the prokaryotic expression plasmid, the PCR products of *recN* and *sspH1*_*1–140*_ from the genomic DNA of *Salmonella* and spliced ATF6 from the cDNA of CT26 cells were cloned into pET28a(+) to express the SspH1:sATF6 fusion protein from the *recN* promoter. The recombinant plasmid was transferred into *Salmonella* strains using electroporation as described previously^58^. For constructing the eukaryotic expression plasmid, the PCR products of sATF6 fused with SspH1 (SspH1:sATF6) from the cDNA of CT26 and *Salmonella* were cloned into pcDNA3.1(+). The recombinant plasmid was transfected into CT26 cells using Lipofectamin 3000 (Invitrogen; Carlsbad, CA, USA).

### Protein extraction and western blot analysis

*Salmonella* was harvested and centrifuged to separate the pellet and the supernatant. The pellets were resuspended in 1 ml PBS, lysed by sonication, and centrifuged to extract the protein. Proteins in the supernatant were precipitated using trichloroacetic acid (TCA) as described previously^59^. Briefly, the sample was mixed with 20% TCA and incubated at 4 °C overnight. The protein solutions were pelleted by centrifugation at 13,000 rpm for 5 min. The protein pellet was washed twice with 200 μl cold acetone and dissolved in PBS.

CT26 cells (1 × 10^6^ cells) were lysed with radio immunoprecipitation assay (RIPA) buffer (Sigma-Aldrich) containing protease inhibitors (Thermo Scientific; Rockford, IL, USA) followed by sonication. The proteins were loaded into each well of a 10% Bolt Bis-Tris gel (Invitrogen), followed by transfer onto nitrocellulose membranes (Bio-Rad). The membranes were blocked with 5% skim milk (BD) in PBS with 0.05% Tween-20 (PBS-T) and incubated with primary antibody (details are listed in Table S5). The probed proteins were visualized using horse radish peroxidase (HRP)-conjugated secondary antibodies (1:5000 dilution; Sigma-Aldrich) and SuperSignal West Pico chemiluminescent substrate (Thermo Scientific). The Bio-rad ChemiDo Touch imaging system (Bio-Rad) was used for data acquisition and analysis.

### Statistical analysis

All data expressed as means ± standard error of mean (SEM) were compared for statistical significance using the Student’s *t*-test or ANOVA (>2 groups) using the GraphPad Prism 6.0 software (GraphPad; San Diego, CA). Mouse survival experiments were analyzed using Kaplan-Meier survival curves and log-rank test of the GraphPad Prism 6.0 software. Differences with *P* values <0.05 were considered significant.

## Supplementary information

Supplementary information.

## Data Availability

The datasets generated during and/or analysed during the current study are available from the corresponding author on reasonable request.
