# Supplementary material for: Development of Oxytolerant Salmonella typhimurium Using Radiation Mutation Technology (RMT) for Cancer Therapy
Source: Sci Rep. 2020 Feb 28;10:3764. doi: 10.1038/s41598-020-60396-6 (PMC7048768; doi:10.1038/s41598-020-60396-6)
Supplement: Supplementary file 1 — Supplementary information. [file 41598_2020_60396_MOESM1_ESM.pdf]

Development of Oxytolerant *Salmonella typhimurium* Using Radiation Mutation Technology (RMT) for Cancer Therapy

Shuang Gao[1,2], Jong-Hyun Jung[1,3], Shun-Mei Lin[1,2], A-Yeung Jang[1,4], Yong Zhi[1,3], Ki Bum Ahn[1], Hyun-Jung Ji[1], Jae Hyang Lim[5], Huichen Guo[6], Hyon E. Choy[2], Sangyong Lim[1,3,*], Ho Seong Seo[1,3,*]

[1]Research Division for Radiation Science, Korea Atomic Energy Research Institute, Jeongeup, Republic of Korea
[2]Department of Molecular Medicine (BrainKorea21 Plus), Chonnam National University Graduate School, Gwangju, Republic of Korea
[3]Department of Radiation Science, University of Science and Technology, Daejeon, Republic of Korea
[4]Department of Internal Medicine, Korea University College of Medicine, Seoul, Republic of Korea
[5]Department of Microbiology, College of Medicine, Ewha Womans University, Seoul 07985, Republic of Korea
[6]State Key Laboratory of Veterinary Etiological Biology, National Foot and Mouth Disease Reference Laboratory, Lanzhou Veterinary Research Institute, Chinese Academy of Agricultural Sciences, Lanzhou, China

*** Corresponding. author:**
Ho Seong Seo
Biotechnology Division, Korea Atomic Energy Research Institute, Jeongeup 56212, Republic of Korea. Phone: +82-63-570-3140; Fax: +82-63-570-3238; E-mail: hoseongseo@kaeri.re.kr
Sangyong Lim
Biotechnology Division, Korea Atomic Energy Research Institute, Jeongeup 56212, Republic of Korea. Phone: +82-63-570-3141; Fax: +82-63-570-3238; E-mail: saylim@kaeri.re.kr

Contents:
Supplementary Methods
Supplementary Figures (S1, S2, S3, S4 and S5)
Full length blots for each Figure
Supplementary Tables (S1, S2, S3, S4 and S5)

Supplementary Methods

Cell viability analysis.

CT26 cells (8×10^3 cells/well) were cultured in 96-well plates (eight replicate wells), followed by transfection with the plasmids and incubated for 5, 24, 48, and 72 h at 37°C in the presence of 5% CO_2. Reagents of the cell counting kit-8 (Dajindo Laboratories; Kumamoto, Japan) were added at the above time points and cultured for another 1–4 h. The absorbance values at 450 nm were read on a Victor™ X3 2030 multi-label reader (PerkinElmer; Waltham, MA, USA).

Cell apoptosis analysis.

CT26 cells were seeded on 6-well plates at a density of 1×10^6 cells/well and transfected with the plasmids. Forty-eight hours after transfection, the cells were collected and washed twice with cold PBS and then resuspended in 100 µl DMEM with 1% FBS. The cells were then incubated with 10 µl each of annexin V and propidium iodide (PI) for 20 min at room temperature in the dark, followed by analysis using the Muse™ cell analyzer within 1 h according to the manufacture's instruction (EMD Millipore Bioscience; Darmstadt, Germany).

Quantitative reverse transcription polymerase chain reaction (qRT-PCR).

Total RNA from *Salmonella* strains or CT26 cells was isolated using the Trizol reagent (Invitrogen) and purified using the RNeasy kit (Qiagen; Hilden, Germany). RNA concentration was measured using a nano photometer (Implen; Minich, Germany) according to the manufacturer's instructions. cDNAs were synthesized from 1.0 µg RNA using PrimeScript™ first strand cDNA synthesis kit (Takara Bio Inc.; Shiga, Japan) according to the manufacturer's instructions. Primers for qRT-PCR were designed using the Primer Express v2.0 software and are shown in Table S4. qRT-PCR amplification was performed using SYBR Premix EX Taq (Takara Bio Inc.) on a Bio-Rad CFX Connect real-time system (Bio-Rad; Hercules, CA, USA). Relative quantification of gene expression was determined using the comparative threshold (ΔΔCr) method. β-actin was used as the control to normalize expression levels.

Supplementary Figures

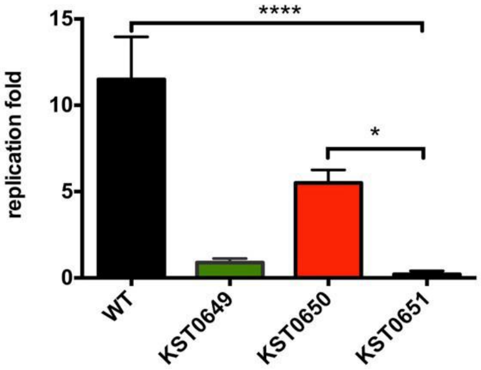

Fig. S1. KST0650 has higher intracellular replication ability in CT26 cell. CT26 cells were infected with WT, KST0649, KST0650 or KST0651 strains at MOI=10 and intracellular bacterial number was enumerated and changes of relative *Salmonella* replication fold were analyzed by the ratio of each *Salmonella* strain in CT26 cells at 18 h and 2 h post-infection. Data are represented as mean ± SEM from the three independent experiments, asterisks (*) indicates a significant difference between each group. (*, $P<0.05$; ****, $P<0.0001$.)

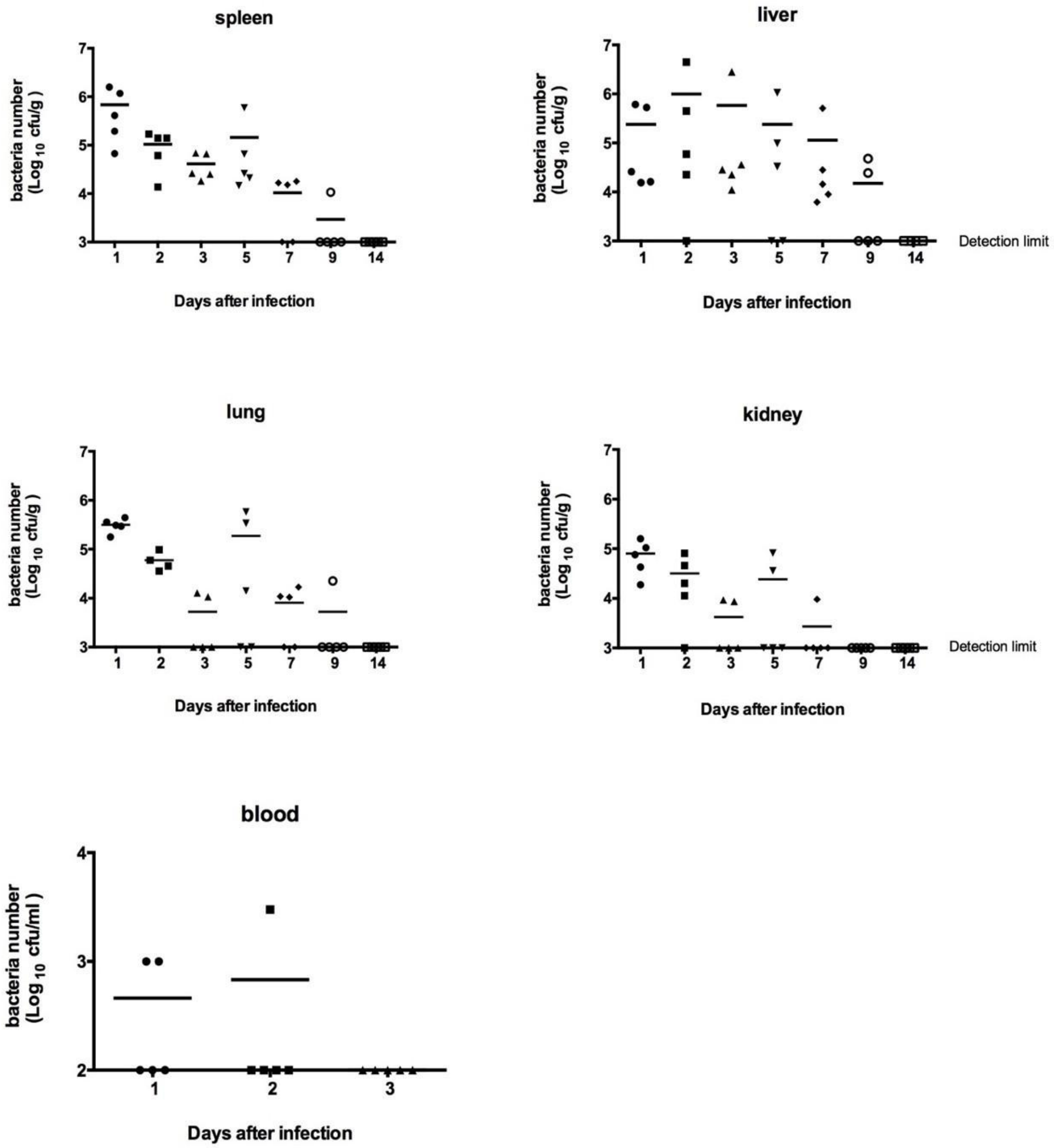

Fig. S2. Clearance of KST0650 in tumor bearing mice. CT26 cancer cell bearing mice were injected i.p. with 1×10^6 cfu KST0650. The numbers of KST0650 in normal tissues of BALB/c mice were calculated for 14 days.

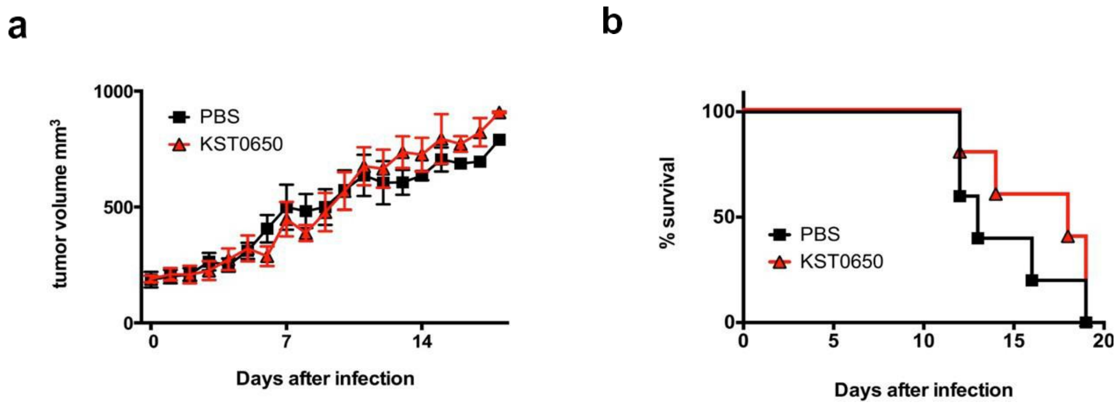

Fig. S3. Suppression of CT26 tumors by KST0650. CT26 cancer cell bearing BALB/c mice were injected i.p. with 1×10^6 cfu KST0650. **(A)** CT26 tumor sizes on BALB/c mice were measured after PBS or KST0650 infection. **(B)** Survival of CT26 tumor bearing mice was monitored for 20 days after PBS or KST0650 infection.

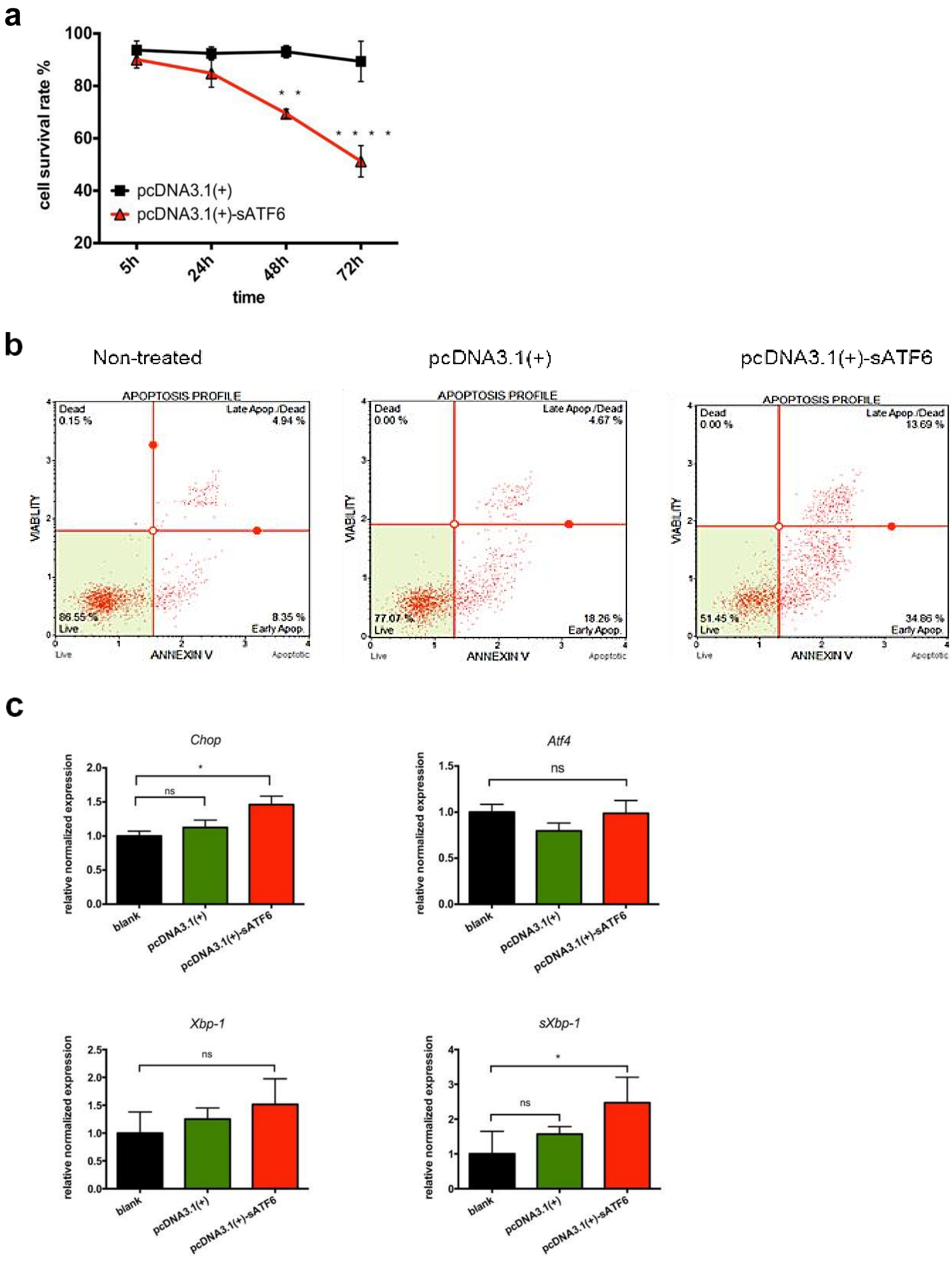

Fig. S4. Apoptotic effect of sATF6 overexpression in CT26 cancer cell. CT26 cell monolayers were transfected with pcDNA3.1(+) or sATF6 expressing vector pcDNA3.1(+)-sATF6. **(A)** sATF6 overexpression inhibited CT26 cell proliferation. Relative cell proliferations were

measured by CCK-8 assay. **(B)** sATF6 overexpression induced CT26 apoptosis and necrosis. Apoptotic and necrotic rates of CT26 cell were determined at 48 h after transfection using Annexin V binding-7-AAD staining assay using Muse™ Cell Analyzer. **(C)** sATF6 overexpression induced Chop and sXbp-1 mRNA expression in CT26 cell. ERS related genes *Chop*, *Atf4*, *Xbp1* and *sXbp-1* expression levels in CT26 cells were evaluated by quantitative real -time PCR at 48 h after transfection. Values were normalized to amount of β-actin gene. Data are represented as mean ± SEM from the three independent experiments and asterisks (*) indicates a significant difference between each group. (ns, no significant; *, P<0.05; **, P<0.01; ****, P<0.0001.)

Full length blots for each Figure

Fig.3b

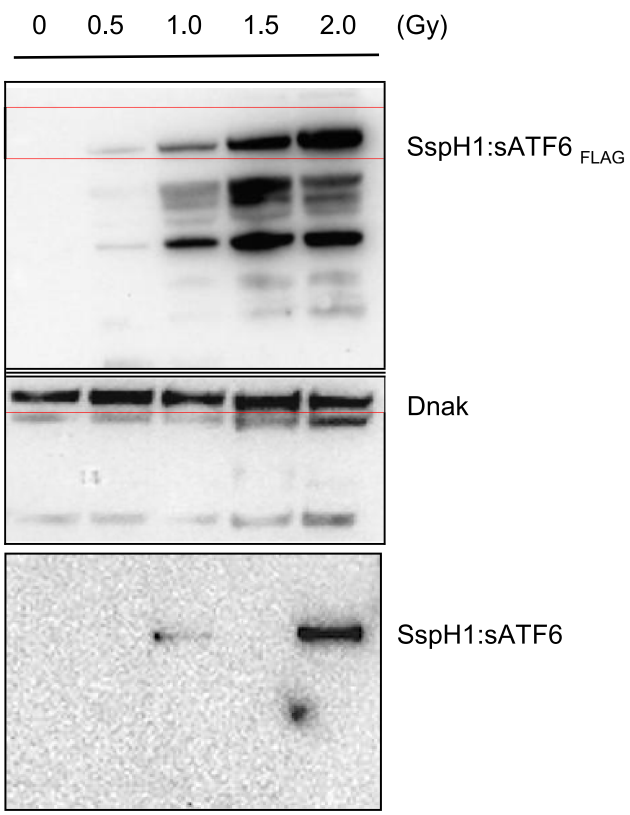

Fig.3c

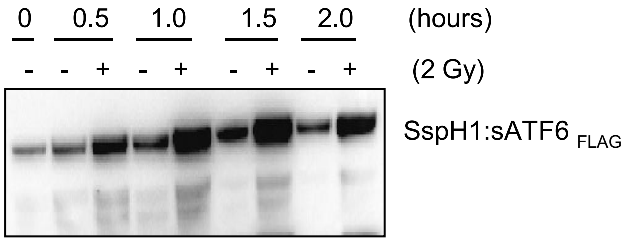

Fig.4a

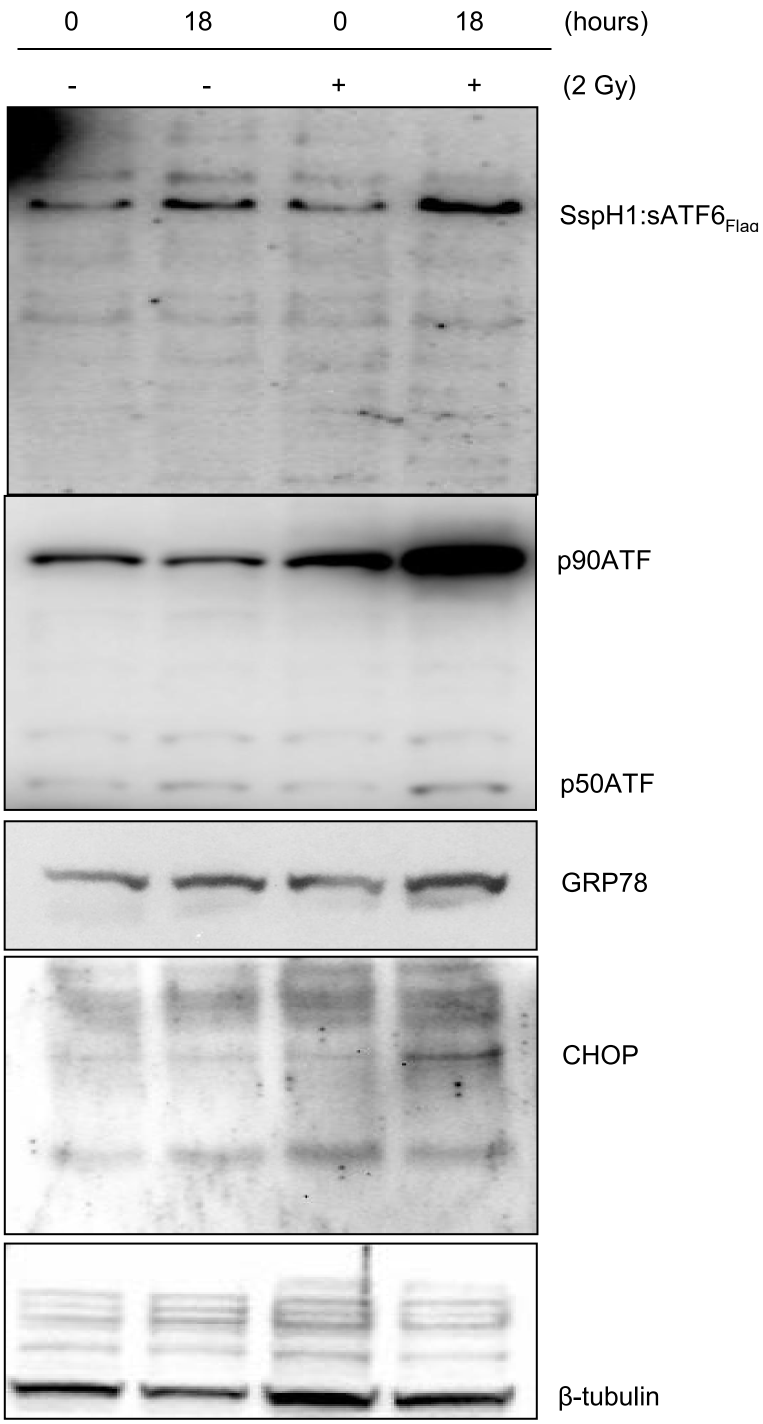
0 18 0 18 (hours)
- - + + (2 Gy)
SspH1:sATF6Flag
p90ATF
p50ATF
GRP78
CHOP
β-tubulin

Supplementary Tables

Table S1. LD_{50} of WT, KST050 and KST0651 in BALB/c mice. BALB/c mice (n=4) were injected i.p. with indicated number of *Salmonella* and monitored their survival for 7 days

	**5×10^{8} CFU**	**1×10^{8} CFU**	**2×10^{7} CFU**	**4×10^{6} CFU**	**8×10^{5} CFU**	**1.6×10^{5} CFU**	**3×10^{4} CFU**
**WT**	0/4	0/4	0/4	0/4	0/4	0/4	1/4
**KST0650**	0/4	0/4	2/4	4/4	4/4	4/4	4/4
**KST0651**	0/4	0/4	0/4	4/4	4/4	4/4	4/4

Table S2. Location of mutations in KST0650 genome

	Gene ID	Gene Name	Position	LT2	KST0650	Mutation	Type	Function
1		NCR[†]	28092	AGGCCTAGG	AGG	deletion	-	
2		NCR	32469	T	G	transversion	-	
3	PSLT104	traD	86180	GCCGCAACAGCCGCAACAGCC	GCC	deletion	Deletion	ATP-binding protein/DNA transporter
4	STM0153	aceF	180034	CG	GC	inversion	Non-sense	dihydrolipoamide acetyltransferase
5	STM0157	yacH	184305	CG	GC	inversion	Mis-sense	putative outer membrane protein
6		NCR*	220824	GC	GCC	insertion	-	
7		NCR	288835	C	T	transition	-	
8		NCR	288843	C	A	transversion	-	
9		NCR	288853	C	T	transition	-	
10	STM0249	rrsH (NCR)	290717	C	A	transversion	-	16S ribosomal RNA
11	STM0395	sbcC	448377	A	G	transition	Silent	ATP-dependent dsDNA exonuclease
12	STM0399	brnQ	453938	C	T	transition	Silent	branched-chain amino acid transporter
13		NCR	510550	G	GC	insertion	-	
14		NCR	520411	GC	GCC	insertion	-	
15	STM0547	fimH	608856	G	C	transversion	Mis-sense	minor fimbrial subunit
16	STM0627	dcuC	691691	C	T	transition	Mis-sense	dicarboxylate transporter
17	STM0682	nagC	742049	G	A	transition	Silent	N-acetylglucosamine repressor, NagC
18	STM0835	STM0835	902371	G	A	transition	Mis-sense	iron (metal) dependent repressor, DtxR family
19	STM0845	moeB	915161	G	A	transition	Mis-sense	[molybdopterin synthase] sulfurylase
20	STM0939	STM0939	1016587	CG	GC	inversion	Mis-sense	hypothetical protein

21	STM1043	STM1043	1129916	GCGCCTAGGTGCT TGGCG	GCG	deletion	Deletion	Gifsy-2 prophage attachment and invasion protein homolog
22	STM1124	putA	1208418	GCC	GCCC	insertion	Frame-shift*	amino acid dehydrogenase
23		NCR	1256605	GC	GCC	insertion	-	
24	STM1280	yeaL	1357016	CG	CGG	insertion	Frame-shift	putative inner membrane protein
25	STM1285	yeaG	1363418	GCCCCCCCCC	GCCCCCCCCCC	insertion	Frame-shift*	putative serine protein kinase, PrkA
26		NCR	1430155	AC	ACC	insertion	-	
27		NCR	1435558	TG	T	deletion	-	
28	STM1425	mdtK	1503292	CG	GC	inversion	Mis-sense	multidrug efflux protein NorA
29	STM1444	slyA	1520176	CG	GC	inversion	Mis-sense[a]	transcriptional regulator SlyA
30	STM1462.S	ydgJ	1536627	CG	GC	inversion	Mis-sense	putative oxidoreductase
31	STM1468	fumA	1543815	CGG	CG	deletion	Frame-shift*	fumarase, class I, homodimeric (EC 4.2.1.2)
32		NCR	1554186	GC	GCC	insertion	-	
33		NCR	1670337	TCC	TCCC	Insertion	-	
34	STM1626	trg	1715973	CG	GC	inversion	Mis-sense	methyl-accepting chemotaxis sensory transducer with TarH sensor
35	STM1702	rnb	1795046	G	A	transition	Non-sense	exoribonuclease II
36		NCR	1841404	G	A	transition	-	
37	STM1753	hnr	1849214	G	A	transition	Non-sense	response regulator
38	STM1753	hnr	1849648	A	C	transversion	Mis-sense	response regulator
39	STM1805	fadR	1906120	C	T	transition	Silent	transcriptional regulator, GntR family
40	STM1828	yoaE	1926893	CGCG	CGCGGCG	insertion	Insertion	putative inner membrane protein
41	STM2107	wcaH	2191593	GCC	GCCC	insertion	Frame-shift*	GDP-mannose mannosyl hydrolase
42	STM2115	wcaA	2198183	C	T	transition	Mis-sense	putative glycosyl transferase
43	STM2194	yeiG	2292159	CG	CGG	insertion	Frame-shift	putative esterase

44	STM2318	nuoL	2427979	C	G	transversion	Silent	NADH dehydrogenase subunit L (EC 1.6.5.3)
45	STM2389	fadI	2500654	CG	GC	inversion	Mis-sense	3-ketoacyl-CoA-thiolase
46	STM2425	yfeH	2537941	CG	GC	inversion	Mis-sense[a]	putative Na+-dependent transporter
47	STM2438	STM2438	2550665	CG	GC	inversion	Mis-sense[a]	putative periplasmic protein
48		NCR	2627555	AC	ACC	insertion	-	
49	STM2528	STM2528	2661659	G	C	transversion	Mis-sense	putative dimethylsulfoxide reductase
50		NCR	2682821	CGG	CGGG	insertion	-	
51	STM2553	csiE	2691581	G	T	transversion	Non-sense	transcriptional antiterminator, BglG family
52	STM2553	csiE	2692225	C	T	transition	Silent	transcriptional antiterminator, BglG family
53	STM2589	STM2589	2737591	GCC	GCCC	insertion	Frame-shift	host specificity protein-J-like
54	STM2609	STM2609	2757548	CG	CGG	insertion	Frame-shift	DNA packaging-like protein
55	STM2619	STM2619	2763753	A	T	transversion	Silent	hypothetical protein
56	STM2689	NCR	2834545	GCC	GCCC	insertion	-	pseudogene
57	STM2851	hycC	2996778	CG	GC	inversion	Mis-sense	NADH dehydrogenase subunit N
58	STM2857	hypD	3001700	CGCG	CGCGGCG	insertion	Insertion	Hydrogenase maturation protein HypD
59	STM2971	sdaB	3123348	CG	GC	inversion	Mis-sense	L-serine ammonia-lyase (EC 4.3.1.17)
60	STM2975	fucP (NCR)	3127648	TGGG	TGG	insertion	-	pseudogene
61	STM2996	recC	3154019	CG	GC	inversion	Mis-sense[a]	DNA helicase/exodeoxyribonuclease V, gamma subunit (EC 3.1.11.5)
62	STM3024	yohM	3184542	CCACGACCA	CCA	deletion	Deletion	Ni/Co efflux system
63	STM3057	ubiH	3219132	CG	GC	inversion	Mis-sense	2-octaprenyl-3-methyl-6-methoxy-1,4-benzoquinol hydroxylase (EC 1.14.13.-)/2-octaprenyl-6-methoxyphenol hydroxylase (EC 1.14.13.-)
64		NCR	3253332	AGG	AGGG	insertion	-	

65	STM3145	hybE	3307825	C	T	transition	Mis-sense	Tat proofreading chaperone HybE
66	STM3415	rpoA	3583381	T	C	transition	Mis-sense	DNA-directed RNA polymerase subunit alpha (EC 2.7.7.6)
67	STM3463	STM3463	3613715	AGGG	AGGGG	insertion	Frame-shift*	hypothetical protein
68	STM3500	pckA	3657650	AC	ACGCC	insertion	Insertion	phosphoenolpyruvate carboxykinase
69	STM3513	malQ	3673617	A	C		Mis-sense	4-alpha-glucanotransferase (EC 2.4.1.25)
70	STM3514	malP	3674896	AGCAACGCCGCAAGCTGCGGATTGCACTGTTTAATCCAGC	AGC	deletion	Frame-shift*	maltodextrin phosphorylase
71		NCR	3676853	CAAAAAAAA	CAAAAAAAAA	deletion	-	
72	STM3530	NCR	3694175	CG	CGG	insertion	Frame-shift	pseudogene
73	STM3633	STM3633	3819803	T	C	transition	Silent	transcriptional regulator, LacI family
74	STM3784	STM3784	3984132	T	C	transition	Mis-sense	PTS system unknown substrate IIA component, Gat family (TC 4.A.5.1.4)
75	STM3828.1N	NCR	4031933	GC	GCC	insertion	-	pseudogene
76	STM3913	gppA	4122924	C	A	transversion	Mis-sense	guanosine pentaphosphatase/exopolyphosphatase
77	STM3913	gppA	4122930	T	C	transition	Mis-sense	guanosine pentaphosphatase/exopolyphosphatase
78	STM3974	tatB	4180828	CAA	C	deletion	Frame-shift*	Sec-independent protein translocase TatB
79		NCR	4350855	C	G	transversion	-	
80		NCR	4350856	A	C	transversion	-	
81	STM4167	hemE	4384227	G	A	transition	Mis-sense	uroporphyrinogen decarboxylase (EC 4.1.1.37)
82	STM4211.1N	NCR	4433282	GTC	GTCTC	insertion	Frame-shift	pseudogene

83		NCR	4459985	GC	GCC	insertion	-	
84		NCR	4606271	TGG	TG	deletion	-	
85	STM4393	rpsR	4631400	CG	GC	inversion	Mis-sense	SSU ribosomal protein S18P
86		NCR	4673419	GTT	GTTT	insertion	-	
87	STM4431	STM4431	4675814	GCC	GCCC	insertion	Frame-shift*	putative thiamine pyrophosphate-requiring enzyme
88	STM4447	STM4447	4689233	AG	AGG	insertion	Frame-shift*	putative periplasmic protein
89	STM4577	smp	4832867	CG	GC	inversion	Mis-sense[a]	hypothetical protein

†NCR: non-coding region

[a]Mis-sense = Two amino acids were changed by the mutation

*Frame-shift = protein sequences were truncated by frame-shift

Table S3. Strains and plasmids used in this study

Strain or plasmid	Relevant genotype	Source or reference
Escherichia coli		
**DH5α**	$F^- r^- m^+$ Ø80d*lacZ*ΔM15	Gibco BRL
***Salmonella* Typhimurium**		
**LT2**	Wild type	ATCC*700720
**KST0649**	LT2*ΔptsIΔcrr*	this study
**KST0650**	Irradiated mutant in LT2*ΔptsIΔcrr*	this study
**KST0651**	LT2*ΔrelAΔspoT*	this study
**KST0652**	KST0650 with pRS-sATF6	this study
**Plasmid**		
**pcDNA3.1(+)**	Mammalian expression vector	ThermoFisher
**pcDNA3.1(+)-sATF6**	sATF6 expressing plasmid	this study
**pET28a(+)**	Protein expression vector	Novagen
**pRS**	Replace T7 promoter with RecN promoter	this study
**pRS-sATF6**	sATF6 expressing plasmid under controlling by *recN* promoter	this study

*, ATCC: American Type Culture Collection

Table S4. Primers used in this study

Target gene	Primer name	Primer sequence (5’-3’)
**recN promoter**	3173	TCA GAT CTG AGG TTT ATA TTG ATG AAA C
	5173	TTT CTA GAA GCT GTT TTC CTG TAT GAA
**sspH1**	3168	AAT CCA TGG CTT GAT GCG TGT GTC TGC G
	5174	TTG CGG CCG CTT CTG TGC ATT CCT CTC CCG
**sATF6 cloning**	3176	AGG CGG CCG CAA TGG AGT CGC CTT TTA GTC CGG
	5176	TTC TCG AGT TAT TTA TCG TCA TCG TCT TTG TAG TCA CTT GGG ACT TTG AGC CTC TGG
**sATF6 RT-PCR**	3184	GGA AGC TTC TAT GGA GTC GCC TTT TAG TCC GG
	5184	TTG CGG CCG CGA CTT GGG ACT TTG AGC CTC
**ATF6 RT-PCR**	ATF6-F	TCC TGA ACA GCG AAG TGT TG
	ATF6-R	ACC CAT GAG GTT TCA AGT CC
**Chop RT-PCR**	CHOP-F	CTG GAA GCC TGG TAT GAG GAT
	CHOP-R	CAG GGT CAA GAG TAG TGA AGG T
**Xbp-1 RT-PCR**	Xbp-1-F	TGG CCG GGT CTG CTG AGT CCG
	Xbp-1-R	GTC CAT GGG AAG ATG TTC TGG
**sXbp-1 RT-PCR**	sXbp-1-F	CTG AGT CCG AAT CAG GTG CAG
	sXbp-1-R	GTC CAT GGG AAG ATG TTC TGG

Table S5. Antibodies used in this study

Antibody	Company	Catalog No.
FLAG	Sigma	F1804
ATF6	Santa Cruz	sc-166659
β-tubulin	Santa Cruz	sc-9104
GRP78	Abcam	ab21685
DDIT3	Abcam	ab11419
Dnak	Assay Designs	SPA-880
